# A reservoir computing approach for forecasting and regenerating both dynamical and time-delay controlled financial system behavior

**DOI:** 10.1371/journal.pone.0246737

**Published:** 2021-02-12

**Authors:** Rajat Budhiraja, Manish Kumar, Mrinal K. Das, Anil Singh Bafila, Sanjeev Singh

**Affiliations:** 1 Institute of Informatics and Communication, University of Delhi South Campus, New Delhi, India; 2 Department of Computer Science Engineering, School of Engineering and Technology, Central University of Haryana, Mahendergarh, Haryana, India; Babasaheb Bhimrao Ambedkar University, INDIA

## Abstract

Significant research in reservoir computing over the past two decades has revived interest in recurrent neural networks. Owing to its ingrained capability of performing high-speed and low-cost computations this has become a panacea for multi-variate complex systems having non-linearity within their relationships. Modelling economic and financial trends has always been a challenging task owing to their volatile nature and no linear dependence on associated influencers. Prior studies aimed at effectively forecasting such financial systems, but, always left a visible room for optimization in terms of cost, speed and modelling complexities. Our work employs a reservoir computing approach complying to echo-state network principles, along with varying strengths of time-delayed feedback to model a complex financial system. The derived model is demonstrated to act robustly towards influence of trends and other fluctuating parameters by effectively forecasting long-term system behavior. Moreover, it also re-generates the financial system unknowns with a high degree of accuracy when only limited future data is available, thereby, becoming a reliable feeder for any long-term decision making or policy formulations.

## Introduction

Ever since its beginning, stock markets and financial systems have always played a defining role in chartering new growth trajectories and shaping economies worldwide. Being such a critical influencer, gaining deep insights by dissecting through historical data and international market behaviors has long been a topic of interest. Once understood, the next step would always have been to judiciously forecast the underlying economic data features, attributed with highly volatile characteristics. By being able to achieve this gives a head-start and unique competitive edge over other market participants. Moreover, these systems display erratic behavior and are highly sensitive [1, 2] to both their initial and prevalent local conditions related to macroeconomic policies, trends, social scenarios, and sentiments. As a result, the global data is fragmented into smaller data sets with each having a unique and segmented behavior of its own. With this additional challenge in the form of limited data, machine learning techniques appear as logical and practical candidates to predict and forecast such chaotic financial systems having non-linear relationships.

Machine learning techniques are available in plenitude with one sizing over the other contingent to the application, or, problem in hand. Having close resemblance to biological brain, artificial neural networks (ANN) have a widespread applicability in almost every domain ranging from medical imaging [3], to healthcare [4], internet-of-things [5] to sports [6] and many more. The advent of error backpropagation [7] and its usage as part of ANN training had been a breakthrough moment which leap-frogged it in becoming the standard training technique for feed-forward neural networks. Applying the same to recurrent neural network (RNN) training met only limited success, since the backpropagated gradients either exploded or vanished over multiple time steps [8]. The cyclic dependencies further left the conventional RNN training techniques non-converging [9] and it required heavy computation to even make them converge, but still this was very slow and led to a problem of local minima. As a result, training recurrent neural networks (RNN) appeared a highly challenging task.

This re-kindled the interest and necessitated for newer training approaches for RNN. Extensive work based on echo state network (ESN) by Jaeger [10, 11] and liquid state machines (LSM) [12] from computational neuroscience started to flow and the stream became collectively known as ‘reservoir computing’. As demonstrated by Lukoševičius & Jaeger [13], this approach employs RNN at the core and the deployed cyclic network in-turn is termed as the “reservoir”. It allows the RNN to perform exceptionally well [14] as the reservoir connection weights are randomly generated and requires only the supervised readout from the reservoir to be trained.

Such an approach which requires training for only a limited part of the network architecture, is computationally fast and at the same time require less resources. This makes it apt for forecasting in chaotic dynamical systems which are very challenging to model and highly sensitive to initial conditions. Even an infinitesimally small change at their start-up results in a completely different system behavior which qualifies its expected non-linear characteristics. Financial systems are a straight-fit into this scenario owing to their dependency on varying parameters which themselves are truly volatile. Various mathematical models exist in practice, but their accuracy is limited to short-term forecasting based on limited data availability and non-linearity in trend relationships. A comprehensive review and comparative performance evaluation for various machine learning techniques has been presented by Ryll and Seidens in [15] where recurrent neural networks are found to out-perform other feed-forward models and machine learning techniques while exploiting the dependencies in financial time series.

In this paper, we consider the task of predicting/forecasting long-term behavior and varying trends of a non-linear financial system using a software-driven reservoir computing approach. The model is constructed taking into consideration that only partial set of values are available for any one of the trend parameters, and the time-synchronized values for remaining trend parameters can be successfully predicted. This follows from the principle of Observability as explained for non-linear dynamical systems by Lu et al. [16] and Hermann & Krener [17].

## Reservoir computing

Reservoir Computing is a highly attractive paradigm and dynamical machine learning approach used in RNN training. It follows the underlying echo state principles, which have recently been successfully applied over differing applications in sentiment analysis [18, 19], health care [20], adaptive control [21], robotics [22, 23], speech recognition [24, 25], financial forecasting [26, 27], and numerous other time series prediction [28, 29] scenarios.

**Fig 1:**
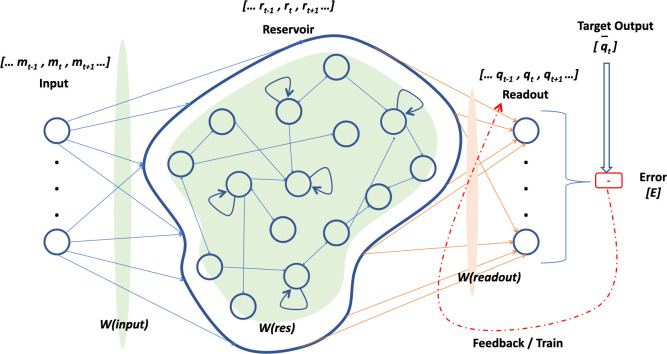
Basic anatomy of recurrent neural network.

Fig 1 shows the basic anatomy of a recurrent neural network. A high-level view showcases RNN to comprise of three distinct layers of connected neurons. The first one serves as a linear input layer for time-dependent multi-dimension inputs denoted by *m(t)*. As with weighted synaptic connection paths, the information is then passed through to second layer, which is a pool, or, reservoir of neurons and are cyclically connected. This hidden reservoir denoted by *r(t)*, is dynamic in nature and is expected to absorb the temporal characteristics from arriving inputs and project those into a high-dimensional reservoir state space which enhances separability. This internal pool is in turn connected to the multi-dimensional output (readout) layer, denoted by *q(t)*, which completes a basic recurrent neural network. Since it is said to follow the echo state property [10, 14], it guarantees the fading away of input influence on the internal reservoir states.

Contrary to other neural networks which involve a bi-directional weight and error update for all the involved layers in succession, reservoir computing adopts a very light-weight strategy which standardizes the input and hidden (reservoir) layer weights. This only leaves the necessity to compute and train the readout layer weights, with the reservoir now behaving as a non-autonomous dynamical system. Such reservoir network characterization plays a defining role in the generated model behavior, along with desired optimizations on non-functional aspects like speed and computational. Owing to its simplicity and low computation cost reservoir computing ranks very high in the list of preferred approaches for RNN training.

## Financial system

Financial modelling and economic dynamics have gained acceleration over the last decade. To gain complete insights and control over core features of economic data exhibiting unpredictable micro- and macroeconomic fluctuations, structural and statistical changes, erratic growths and coinciding streams of development—is a strenuous ask. Such irregular behavior is often attributed to external agents influencing the system, but still, researchers have invested considerable effort and have come up with stochastic analysis and non-linear characterization for such systems which are very essential from an economic perspective. Following pursuit, we consider an extremely challenging and a non-linear chaotic finance model which involves interest rate, investment demand and price index as the underlying moving parameters. As presented by Ma and Chen in [30, 31], such model can be described in terms of a dimensionless system of first order coupled linear differential equation in Eq 1 below.

$$\dot{X}=Z+(Y-s)X$$

$$\dot{Y}=1-cY-X^{2}$$

$$\dot{Z}=-X-eZ$$ (1)

where, X—interest rate, Y—investment demand, Z—price index, s—saving amount, c—cost per investment, e—elasticity of demand in commercial markets.

In general, such a system displays complex chaotic behavior as demonstrated by Chen [32] and Jain & Das [33]. The system is shown to have 3 equilibrium points EP_1_, EP_2_ and EP_3_ which are either asymptotically stable, or, saddle depending on initial conditions mentioned in Eq 2.

EP1:x0=0,y0=1c,z0=0 (2)

EP2,3:x0=±(e-c-sce)e,y0=1+see,z0=∓1e(e-c-sce)e

Having dependency on forces external to the system under consideration, it becomes extremely important to have some methodology in-place through which such chaotic dynamics can be controlled. By introducing a time-delayed feedback as showcased by Chen [32] and Pyragas [34], such complex dynamical systems can be successfully transitioned to exhibit regular dynamics, thereby, make them tractable with widespread applicability in policy decision making and economic forecasting. The updated model having time-delay parameters [32–34] can be then represented as in Eq 3.

$$\dot{X}=Z+(Y-s)X+f_{1}(X-X(t-\tau_{1}))$$

$$\dot{Y}=1-cY-X^{2}+f_{2}(Y-Y(t-\tau_{2}))$$

$$\dot{Z}=-X-eZ+f_{3}(Z-Z(t-\tau_{3}))$$ (3)

where, *f*_*i*_–feedback strength and *τ*_*i*_–time delay for *i* = 1, 2, 3. Proper tuning for feedback strength and time-delay parameters, easily ratified using bifurcation diagrams as in [33] is shown to have a stabilizing influence, and, hence induces a regular and highly controlled system behavior.

Basis the above, it’s evident that selection of an appropriate modelling technique is highly dependent on the data characteristics and patterns originating from the observed system. Theoretical modelling techniques can be easily applied to scenarios displaying regular and periodically aligned data. On the other hand, machine learning techniques gain much better ground and are better suited to handle non-linear, complex data owing to their inherent capability to learn, self-update and re-learn cyclically. The next section illustrates the specific setup, design implementation and procedures for modelling the afore-mentioned financial system.

## Data and procedure

In our present work, we consider the financial system as detailed above. Since it exhibits both chaotic and regular behavior characterizations, we thought of using a common technique capable of addressing both the system behavior scenarios simultaneously. From the plethora of available approaches, we have restricted ourselves in using reservoir computing technique, since it is known to perform exceedingly well on non-linear time-series data irrespective of the application domain. Though it appears straightforward and conceptually simple, still it needs to be carefully implemented for achieving good performance figures. Lukoševičius [35] has presented a comprehensive view on efficiently configuring the reservoir. The intent of this work is not to validate the authenticity of the discussed financial model, but instead, to predict its behavior correctly using the reservoir computing approach. This section thoroughly details the internals for configuring the light-weight reservoir computing setup along with its step-wise processing and updates with respect to the complex input data.

### Data-sets

As previously discussed, we use a financial system to derive the targeted model. Two input time series are generated corresponding to Eqs 1 and 3 respectively, while using the initial conditions *X*_*0*_ = 2.0, *Y*_*0*_ = 3.0 and *Z*_*0*_ = 2.0; along with control parameters as deduced by [33] and captured in Table 1. As part of current work, only feedback strength *f*_1_ is tuned, while retaining *f*_2_ and *f*_3_ as 0. The first time-series corresponds to complex chaotic data with the system displaying non-linear behavior, while the second one reflects a controlled and regular behavior for the same financial system acting under the influence of a properly configured time-delayed feedback. Henceforth, these two time-series inputs will be referred to as “ChaoticInput1” and “ControlledInput2”.

**Table 1:** Control parameters for financial system.

Control parameter	Value
**Saving amount (*s*)**	3.0
**Cost per investment (*c*)**	0.1
**Elasticity of demand (*e*)**	1.0
**Feedback strength (*f***_**1**_**)**	0.1
**Feedback strength (*f***_**2**_**)**	0.0
**Feedback strength (*f***_**3**_**)**	0.0
**Delay parameter (*τ***_**1**_**)**	5.0
**Delay parameter (*τ***_**2**_**)**	0.00001
**Delay parameter (*τ***_**3**_**)**	0.00001

### Dimensioning the reservoir

To be truly efficient, the derived model needs to be agnostic to input values and at the same time be able to service multi-dimensional temporal inputs and outputs. So, our ESN-based reservoir computing setup comprises a M-dimensional input, *m(t)*, forming the input layer which is internally fed to a R-dimensional cyclic reservoir network, *r(t)*, (also called the hidden network layer). This hidden layer, in turn, is connected to a Q-dimensional output, *q(t)*, which forms the readout/output layer. As is the case with any neural network, the layer dimensionality has an analogous mapping to number of neurons, implying M neurons in the initial input layer, R neurons forming the reservoir and Q neurons as part of the final readout layer. The efficiency of any neural network driven model is in principle governed by the number density of neurons and synaptic connection weights configured between them. For the above mentioned topology based on networks defined by Jaeger [10] and Lu [16], we have an input connection matrix *W*^*input*^ with size *R* × *M*, an internal reservoir connection matrix *W*^*res*^ with size *R* × *R*, and, an output readout connection matrix *W*^*readout*^ with size *Q* × *R*. Certain scenarios may require a feedback connection matrix *W*^*fb*^ with size *R* × *Q*, as well, since it helps in providing the (error) differential back from outputs to readout layer while training the reservoir.

The setup presented in this paper was designed and implemented in MATLAB. Our implementation considers a step-wise update *s*(*t*) for the reservoir states having size *R X Q*, where each step maps to the pre-defined time instants for training data-set. Mathematically, this step-wise update for next RNN state is a function of existing state, existing output and available inputs represented by function *G* in Eq 4.

s(t+1)=G(s(t),q(t),m(t+1)) (4)

A more explicit form of this RNN step-update equation derived from [16, 36, 37] can be written as Eq 5.

$$s(t+1)=(1-\alpha ).s(t)+\alpha .H(W^{input}.m(t+1)+W^{res}.s(t)+W^{fb}.q(t)+\eta I)+\phi I$$ (5)

where, *α* is the leaking rate of the reservoir which helps in regulating the speed of reservoir update dynamics. A value of α → 1 fastens the reservoir evolution, whereas, *α* → 0 renders it to update slowly; *H* is an activation function, preferably a non-linear function since it helps in attaining saturation for large values; *ηI* is a noise term which helps in immunizing the reservoir against overfitting based on input data; *ϕI* is a constant bias term with *ϕ* as the scalar constant and *I* as unitary matrix. Post the reservoir state update, the temporal output *q*(*t* + 1) is evaluated using Eq 6.

$$q(t+1)=W^{readout}.s(t)$$ (6)

### Connection weight matrices

As for an ESN, the connection weight matrices *W*^*input*^, *W*^*res*^, *W*^*fb*^ are randomly defined and kept fixed since start, based on which the *W*^*readout*^ is derived during training process. A similar procedure as referred by Noah [37], with different tunings is used for generating these matrices. Iterative simulations were done during matrix generation resulting in varying RMSE values, and the choice of finalized weight matrix was based on having the most optimal RMSE value. *W*^*input*^ and *W*^*fb*^ generation involved a similar procedure as detailed below:

1. Initial parameters comprised of density (*d*^*input*^, *d*^*fb*^) *d ϵ* (0,1) and radius *σ*(*σ*^*input*^, *σ*^*fb*^)
2. Create an all-zero matrix having correct dimensions (*R* × *M*) *or* (*R* × *Q*)
3. For each matrix entry (*x*,*y*), draw a random number *c ϵ* (0, 1)
  - If *c* < *d* then matrix entry (*x*,*y*) = *random number l ϵ* (−*σ*,*σ*)
  - If *c* ≥ *d* then matrix entry (*x*,*y*) = 0

A different procedure was used during *W*^*res*^ generation which included following steps:

1. Initial parameters comprised of reservoir radius (*σ*^*res*^)
2. Create an adjacency matrix of correct size (*R* × *R*)
3. For each non-zero matrix entry (*x*,*y*), draw a random number *c ϵ* (0, 1)
  - If *c* < 0.5, then matrix entry (*x*,*y*) = −*σ*^*res*^
  - If *c* ≥ 0.5, then matrix entry (*x*,*y*) = *σ*^*res*^
4. With above steps, we obtain the initial un-scaled matrix *W*^0^
5. Final reservoir matrix *W*^*res*^ is obtained by re-scaling each entry as

$$W^{res}=\frac{\rho }{\left|\lambda^{max}\right|}W^{0}$$

where, *ρ* is spectral density and *λ*^*max*^ is the greatest magnitude eigenvalue of *W*^0^.

MATLAB functions *rand()* and *sprand()* have been used for generating random number and adjacency matrix respectively during above procedures.

### Teacher training and testing

Like any model building procedure, this involves teacher training the model for *τ*_1_ reservoir update steps in first leg and testing out the trained model during the remaining part (*t*_2_). If the model is properly trained, then the predicted values must match with expected outputs. In other words, the inputs hold significance during model training, which gradually pass on their impact control to feedback handling during prediction process. This forms the basis of having a similar mechanism for generating both *W*^*input*^ and *W*^*fb*^ weight matrices.

During the training procedure, there is a brief period of transients (also referred to as *dropouts*) beyond which the readouts start displaying the systematic variations corresponding to the training sequence. The present work uses Ridge regression technique described using Eq 7 for evaluating the weights of the readout connection matrix *W*^*readout*^. Here, *β* is the regularization constant which helps to make the network less susceptible to noise and overfitting [13], and, S, V corresponds to reservoir state update vector and teacher training output vector respectively (S and V exclude the dropout samples).

$$W^{readout}=V^{\prime }\;S\;{\left(S^{\prime }S+\beta I\right)}^{-1}$$ (7)

## Experiment and results

Our experiment considers three inputs namely, interest rate (IR), investment demand (ID) and price index (PI) which results in three-dimensional input and output weight matrices. The number of reservoir nodes is one of the most critical hyperparameter, since this number is directly proportional to the overall computation spent on the experiment. There is no fixed value for this, and the optimal size is reached at by running multiple iterations of the experiment while trying out different values. A large reservoir size (more than 1000) is expected to result in better performance, but on the contrary it results in overfitting and requires appropriate regularization. Moreover, such higher values require large computation resources and is generally recommended to make up for scenarios having very limited input training data. Simulation having reservoir size (R) ranging from 50 to 700 nodes, with an incremental increase of 50 nodes at every step was performed. Choice of other parameters like input radius (*σ*^*input*^), reservoir density (*d*^*res*^) and reservoir radius (*σ*^*res*^) having similar values for both experiments is also driven by thorough simulation and analysis based on recommendations [35] for successfully applying ESNs. Finally, based on the entire hyper-parameter set detailed below in Table 2, a suitable value of reservoir size having 400 neurons was determined. Similarly, leaking rate (*α*) was also required to be optimized corresponding to differing activations functions.

**Table 2:** Network hyper-parameters summary.

Hyper-parameter	Experiment set 1	Experiment set 2
**Input dimension (*M*)**	3	3
**Reservoir dimension (*R*)**	400	400
**Readout layer dimension (*Q*)**	3	3
**Input density (*d***^***input***^**)**	0.8	0.5
**Input radius (*σ***^***input***^**)**	0.8	0.8
**Reservoir density (*d***^***res***^**)**	0.5	0.5
**Reservoir radius (*σ***^***res***^**)**	0.5	0.5
**Feedback density (*d***^***fb***^**)**	0.8	0.0
**Feedback radius (*σ***^***fb***^**)**	0.5	0.0
**Reservoir Spectral Radius (*ρ*)**	0.5	0.8
**Leaking rate (*α*)**	0.1/0.2/0.4 ^#^	0.30
**Normalization constant (*β*)**	0.000001	0.000001
**Bias (*ϕ*)**	1.0	0.0
**Noise (*η*)**	0.05	0.0

Note:

1. Selected some discrete values of hyperparameters among many good ones: each parameter seems robust in a quite substantial range of values.

2. ^#^ ChaoticInput1–0.1 for purely linear and non-linear, 0.4 for quadratic and tanh.

^#^ ControlledInput2–0.1 for purely linear and non-linear, 0.2 for quadratic and tanh.

Along with the above defined parameters, the activation function plays a major role during the state vector update stage. Different activation functions like triangler, tanh, mexican hat, sinc have been detailed in [38] for their effects on predictability in echo state networks. It is evident that activation functions with appropriate non-linearity show better performance compared to linear ones and tanh is one of the most extensively used amongst non-linear functions. As in [37], the present work also weighs upon the effect of four different activations functions on the financial system data. The four functions can be broadly categorized as linear and non-linear, with the non-linear category comprising of tanh, quadratic and purely non-linear functions, apart from the strictly linear function. The linear activation function resulting from Taylor’s expansion of tanh(*x*) at *x* = 1 is *f*(*x*) ≈ 0.76159 + 0.41997*x*. Similarly, the quadratic and strictly non-linear activation function are represented by *f*(*x*) ≈ 0.76159 + 0.41997*x* − 0.31958*x*^2^ and *f*(*x*) ≈ 0.76159 − 0.31958*x*^2^ respectively. Configuration related to the teacher training, test data and step update size is mentioned as part of Table 3.

**Table 3:** Dataset distribution.

Input times-series length	Experiment set 1	Experiment set 2
**Teacher training (*t***_**1**_**)**	75	75
**Test length (*t***_**2**_**)**	25	25
**Step size**	0.01	0.01
**Dropouts**	100	100

Utilizing the above hyper-parameter set and dataset distributions, the present study effectively demonstrates and consolidates two different sets of experiments. The first set involves the prediction/forecast of the entire financial time-series based on only feedback-driven reservoir (zero input during test leg), and, the second set involves a partial re-generation of financial time-series based on the generic causal-effect model. These experiments are performed for both the “ChaoticInput1” and “ControlledInput2” datasets.

### Experiment set 1

**Fig 2:**
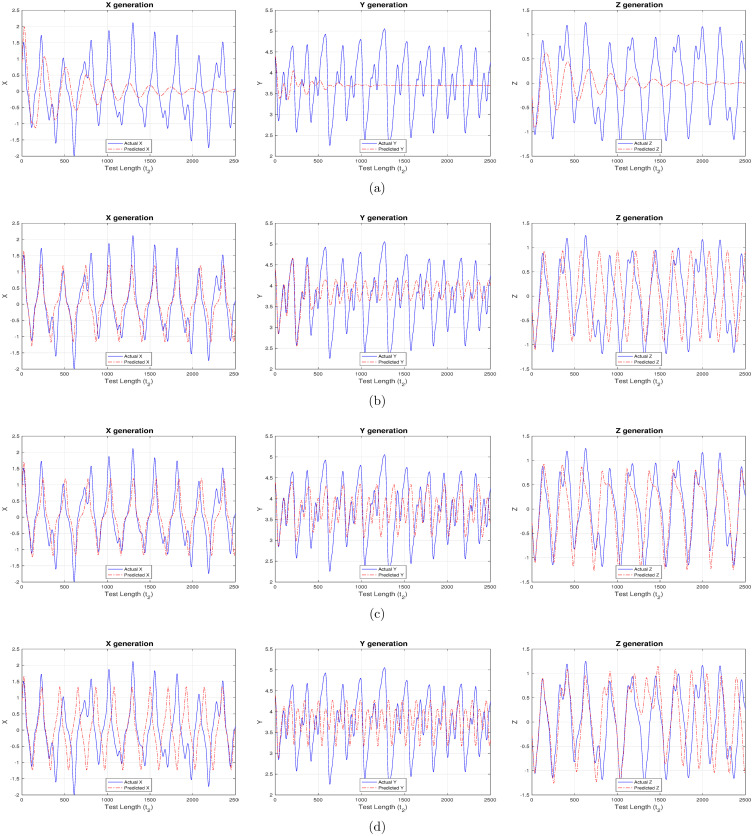
Predicting the chaotic financial system behavior using different activation functions. (a) linear activation (b) strictly non-linear activation (c) quadratic activation (d) tanh activation.

**Fig 3:**
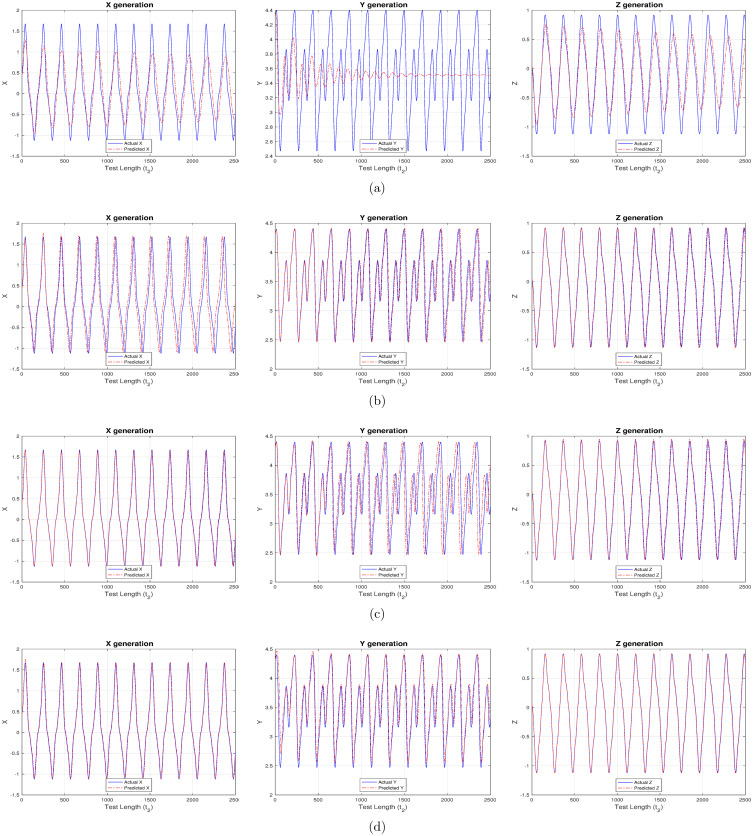
Predicting the controlled (time-delayed feedback) financial system behavior using different activation functions. (a) linear activation (b) strictly non-linear activation (c) quadratic activation (d) tanh activation.

This comprises the most sought-after use case which involves forecasting the irregular capital market, or, financial system behavior. Here in, the entire financial system behavior needs to be predicted based on the trained model and no external input is available post the teacher training period. The model response in forecasting the financial system is completely dependent on how well the reservoir learnt during training and the current output (now being passed as feedback, or, input). Hence, this is referred to as a feedback-only driven system. Figs 2a–2d and 3a–3d showcase predicted system behavior using “ChaoticInput1” and “ControlledInput2” datasets respectively. As evident from Fig 2, the reservoir can predict with a higher accuracy when activation functions have a non-linear component, compared to the strictly ‘linear’ activation function. This is determined based on the trend and overlap between the predicted (red) and actual target (blue) time-series in the figures.

The reservoir retains the system behavior for a good enough time beyond which its memory starts to fade away and deviation is observed. In our case, memory fades much faster when using a purely linear component as in Fig 2a. On the other hand, the models using non-linear components during activation continue to exhibit the expected behavior for a much longer duration of time. Though the predicted values are not exact (reflected as non-overlap in figures), but they could mirror the system behavioral pattern for a prolonged duration as witnessed in Fig 2b–2d. The near conformance in output pattern even ranges for the entire test cycle having 2500 steps for X and Z components as shown in Fig 2b–2d. As observed from experimental results, amongst the non-linear activation functions tanh is seen to perform marginally better than the quadratic and strictly non-linear components. This behavior can be further ratified from Table 4 which contains the mean square error (MSE) values evaluated using Eq 8, where, $E(q,\overset{-}{q})$ refers to error (*E*) between actual $\left(\overset{-}{q}\right)$ and inferred (*q*) readout values, and T is the total steps for which error is evaluated. ‘tanh’ reports the lowest and ‘linear’ the highest MSE values.

$$E(q,\overset{-}{q})=\frac{1}{T}\;\sum_{n=1}^{T}{\left(q_{i}(n)-\overset{-}{q_{i}}(n)\right)}^{2}$$ (8)

**Table 4:** Mean square error (MSE) for chaotic and controlled system in Experiment set 1.

Activation Functions	Chaotic System			Controlled (Time-delayed) System		
	X	Y	Z	X	Y	Z
**Linear**	0.56237	0.43259	0.37167	0.12973	0.26442	0.15067
**strictly Non-linear**	0.27454	0.39703	0.64632	0.20663	0.10347	0.074655
**Quadratic**	0.30308	0.55811	0.23816	0.011126	0.1489	0.06752
**Tanh**	0.39351	0.47391	0.25859	0.010549	0.12472	0.047671

A time-delayed financial system is seen to provide better results both in terms of prediction accuracy and reservoir memory as depicted in Fig 3a–3d. There exists only a minute difference in overlap between predicted and actual outputs, and, additionally this system is seen to predict precisely for a much longer term when compared to system devoid of time-delayed feedback. Apart from the behavioral pattern, the predicted values are a near perfect match to the actual targets while using non-linear components during activation.

This is further corroborated by the very low MSE values in Table 4, where, `tanh’ is again seen to perform the best amongst all activations, thereby making it the preferred option of choice while designing and driving such cyclic reservoirs. The above clearly show the importance of non-linearity while modelling network reservoirs in predicting complex chaotic system behavior.

### Experiment set 2

In addition to direct system forecasting, there may be practical situations when it is not required to fully predict such non-linear systems i.e. limited future data for some components, or, system variables in already available. This necessitates the need to effectively re-generate only the required information based on available data and aligns well with the generic causal effect model, wherein one dependent quantity (say, d_1_) can be derived based on the available temporal values of another (say, d_2_) when, d_1_ and d_2_ have an inter-dependency relationship. Mapping this onto our financial model, reservoir computing approach provides a huge value-add by being able to behave as an input-driven reservoir for the three components IR (or, X), ID (or, Y) and PI (or, Z) one-by-one and the other two components being simultaneously read-out from the system.

**Fig 4:**
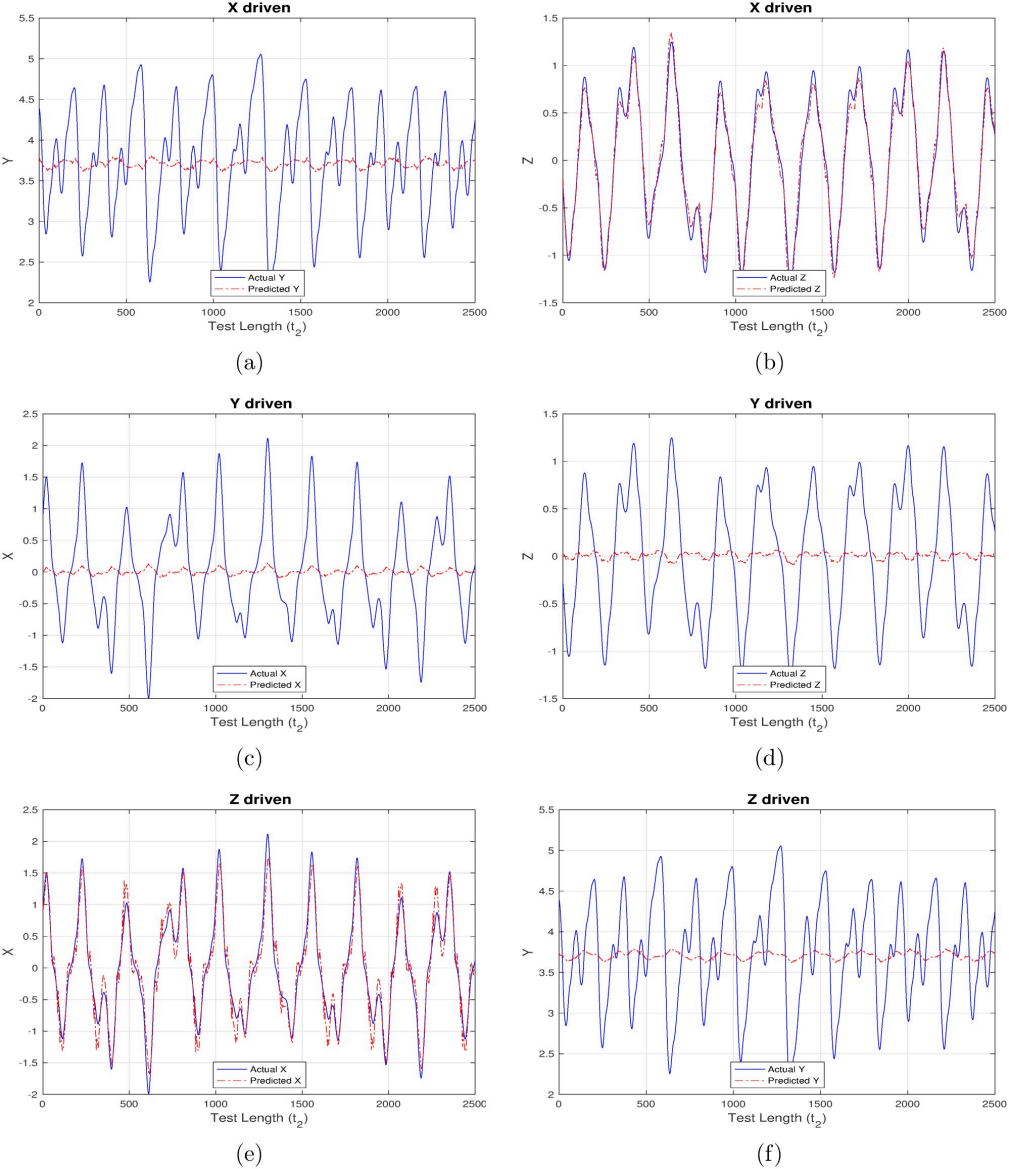
Chaotic financial system component re-generation using “linear” activation. (a) Re-generating “investment demand” [Y] behavior with “interest rate” [X] as input (b) Re-generating “price index” [Z] behavior with “interest rate” [X] as input (c) Re-generating “interest rate”[X] behavior with “investment demand”[Y] as input (d) Re-generating “price index” [Z] behavior with “investment demand” [Y] as input (e) Re-generating “interest rate” [X] behavior with “price index” [Z] as input (f) Re-generating “investment demand” [Y] behavior with “price index” [Z] as input.

**Fig 5:**
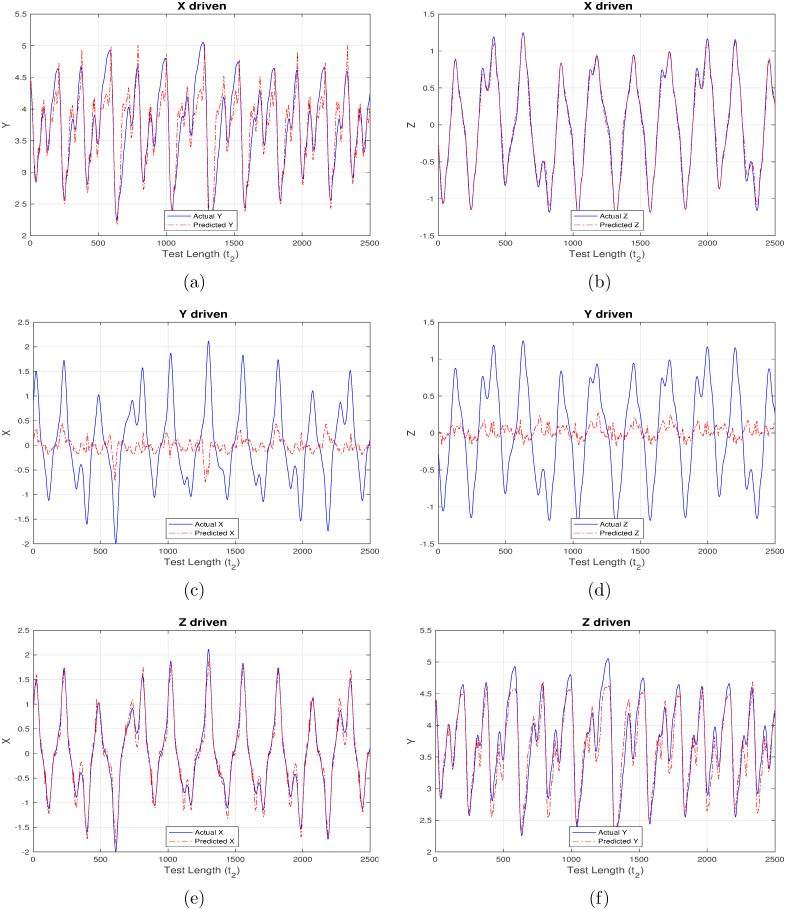
Chaotic financial system component re-generation with “non-Linear” activation. (a) Re-generating “investment demand” [Y] behavior with “interest rate” [X] as input (b) Re-generating “price index” [Z] behavior with “interest rate” [X] as input (c) Re-generating “interest rate”[X] behavior with “investment demand”[Y] as input (d) Re-generating “price index” [Z] behavior with “investment demand” [Y] as input (e) Re-generating “interest rate” [X] behavior with “price index” [Z] as input (f) Re-generating “investment demand” [Y] behavior with “price index” [Z] as input.

**Fig 6:**
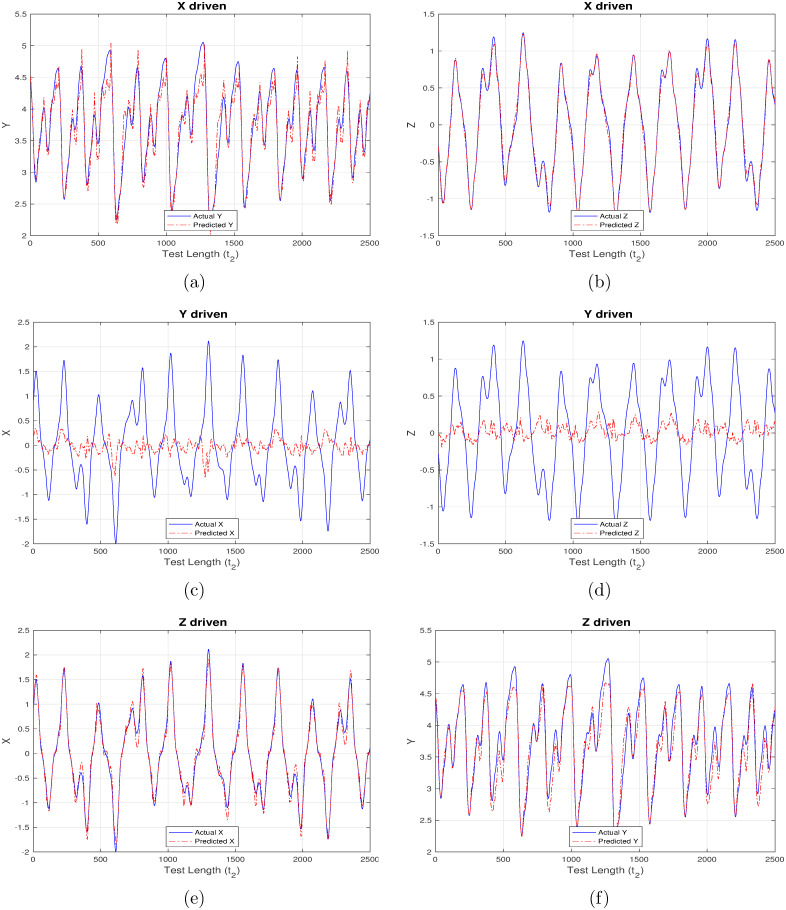
Chaotic financial system component re-generation using “quadratic” activation. (a) Re-generating “investment demand” [Y] behavior with “interest rate” [X] as input (b) Re-generating “price index” [Z] behavior with “interest rate” [X] as input (c) Re-generating “interest rate”[X] behavior with “investment demand”[Y] as input (d) Re-generating “price index” [Z] behavior with “investment demand” [Y] as input (e) Re-generating “interest rate” [X] behavior with “price index” [Z] as input (f) Re-generating “investment demand” [Y] behavior with “price index” [Z] as input.

**Fig 7:**
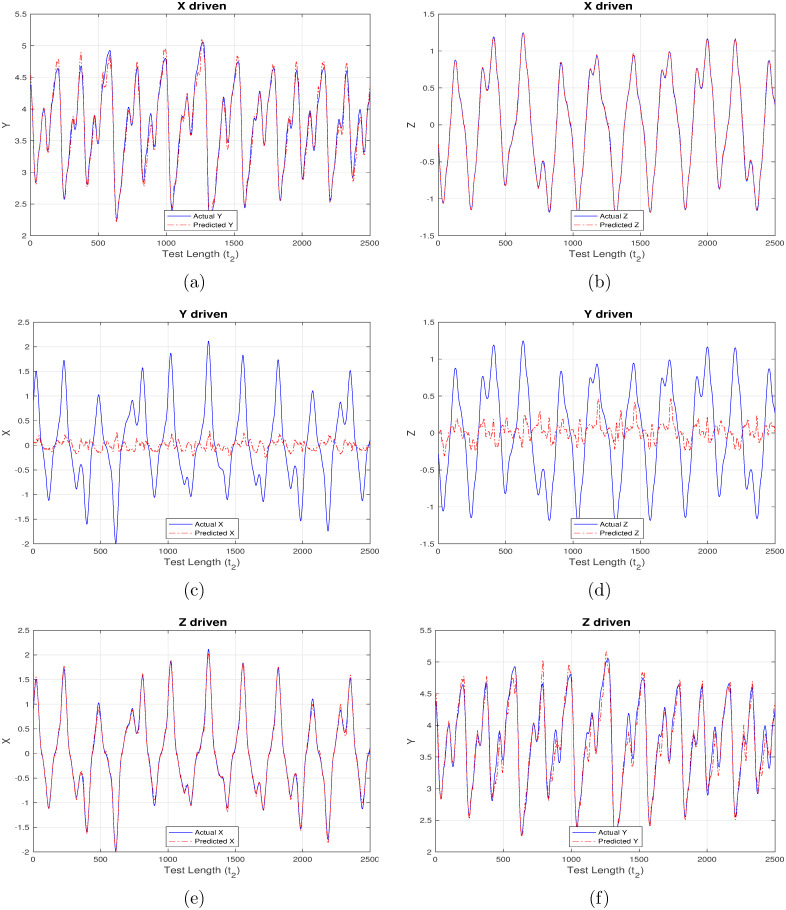
Chaotic financial system component re-generation using “tanh” activation. (a) Re-generating “investment demand” [Y] behavior with “interest rate” [X] as input (b) Re-generating “price index” [Z] behavior with “interest rate” [X] as input (c) Re-generating “interest rate”[X] behavior with “investment demand”[Y] as input (d) Re-generating “price index” [Z] behavior with “investment demand” [Y] as input (e) Re-generating “interest rate” [X] behavior with “price index” [Z] as input (f) Re-generating “investment demand” [Y] behavior with “price index” [Z] as input.

First set of results are generated corresponding to “ChaoticInput1” as shown in Figs 4–7 wherein each figure maps to the usage of separate activation function (Fig 4 –linear activation, Fig 5 –strictly non-linear activation, Fig 6 –quadratic activation and Fig 7 –tanh activation). Further, each figure contains three set of plots, with each set corresponding to one component driving the reservoir and remaining two components being inferred from the model. For example, Fig 4a and 4b contains the plot when X-component (interest rate) is used to drive the reservoir and the remaining components Y (investment demand), Z (price index) are predicted. Similarly, Fig 4c–4f detail the model behavior with Y and Z-component driving the reservoir respectively, and (X, Z), (X, Y) components being predicted respectively.

Even a high-level analysis of Figs 4–7 is enough to conclude that activation functions having a non-linear component clearly out-perform the strictly linear activation function. The linear activation function is found to behave exceedingly well while correlating X and Z components as shown in Fig 4b and 4e. This is deduced owing to a very marginal difference between the actual (blue line) and inferred (red line) outputs i.e. Z component is precisely predicted from X-driven reservoir, and, X component is precisely predicted from Z-driven component respectively. This strong correlation can be attributed to their one-to-one dependence as evident from Eq 1. The introduction of Y component either for driving the reservoir, or, during inference doesn’t generate expected results there by indicating the requirement for a non-linear component which to an extent helps in driving the system behavior towards saturation.

From Figs 5–7 it is observed that the X-driven and Z-driven reservoir system could predict the remaining components to a high degree of accuracy. This is verified by the overlap happening between the actual and inferred components. On the other hand, it is evident that Y-driven reservoirs fail to precisely predict the other components and hence observability doesn’t apply here. Not applicable here, but in certain systems the ambiguity in states may result from the change in signs of X and Z components, as shown by Lu [16] while applying observability over chaotic dynamical systems.

**Fig 8:**
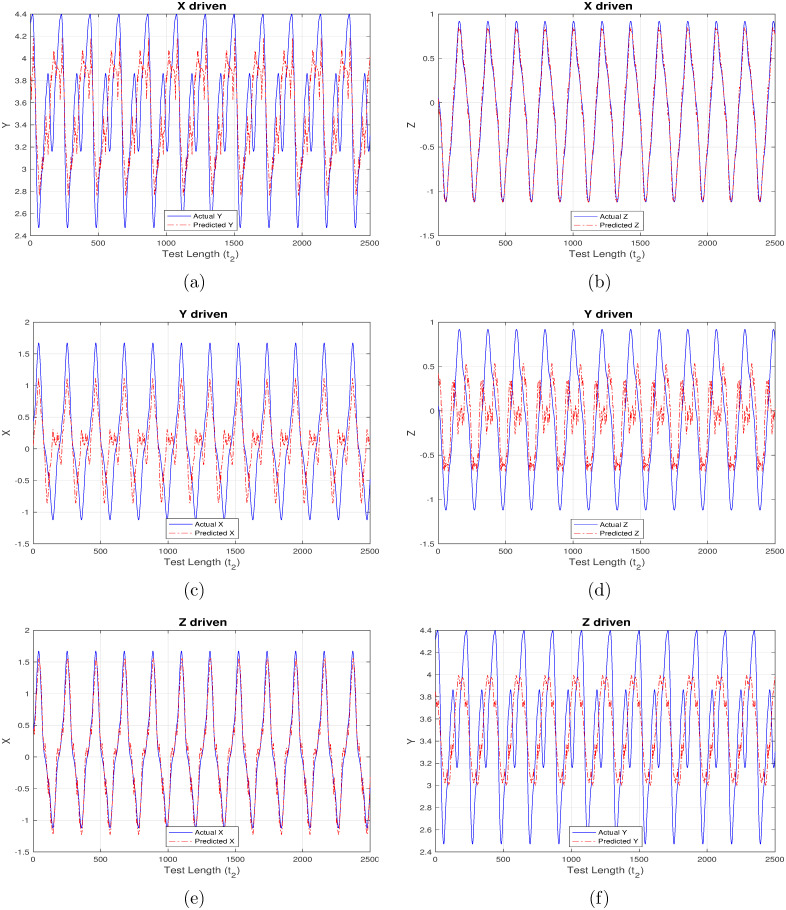
Controlled (time-delayed feedback) financial system component re-generation using “linear” activation. (a) Re-generating “investment demand” [Y] behavior with “interest rate” [X] as input (b) Re-generating “price index” [Z] behavior with “interest rate” [X] as input (c) Re-generating “interest rate”[X] behavior with “investment demand”[Y] as input (d) Re-generating “price index” [Z] behavior with “investment demand” [Y] as input (e) Re-generating “interest rate” [X] behavior with “price index” [Z] as input (f) Re-generating “investment demand” [Y] behavior with “price index” [Z] as input.

**Fig 9:**
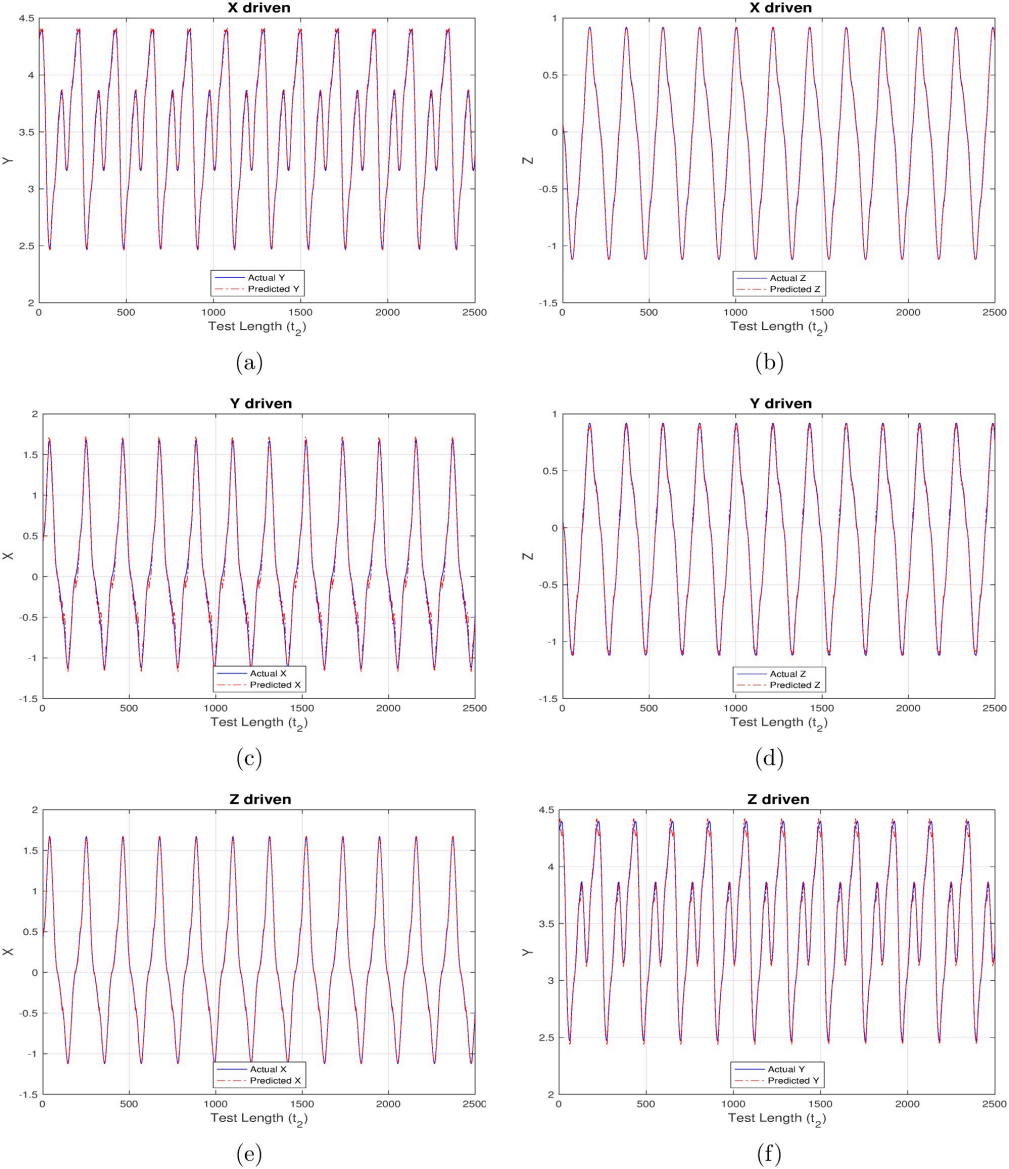
Controlled (time-delayed feedback) financial system component re-generation using “non-linear” activation. (a) Re-generating “investment demand” [Y] behavior with “interest rate” [X] as input (b) Re-generating “price index” [Z] behavior with “interest rate” [X] as input (c) Re-generating “interest rate”[X] behavior with “investment demand”[Y] as input (d) Re-generating “price index” [Z] behavior with “investment demand” [Y] as input (e) Re-generating “interest rate” [X] behavior with “price index” [Z] as input (f) Re-generating “investment demand” [Y] behavior with “price index” [Z] as input.

**Fig 10:**
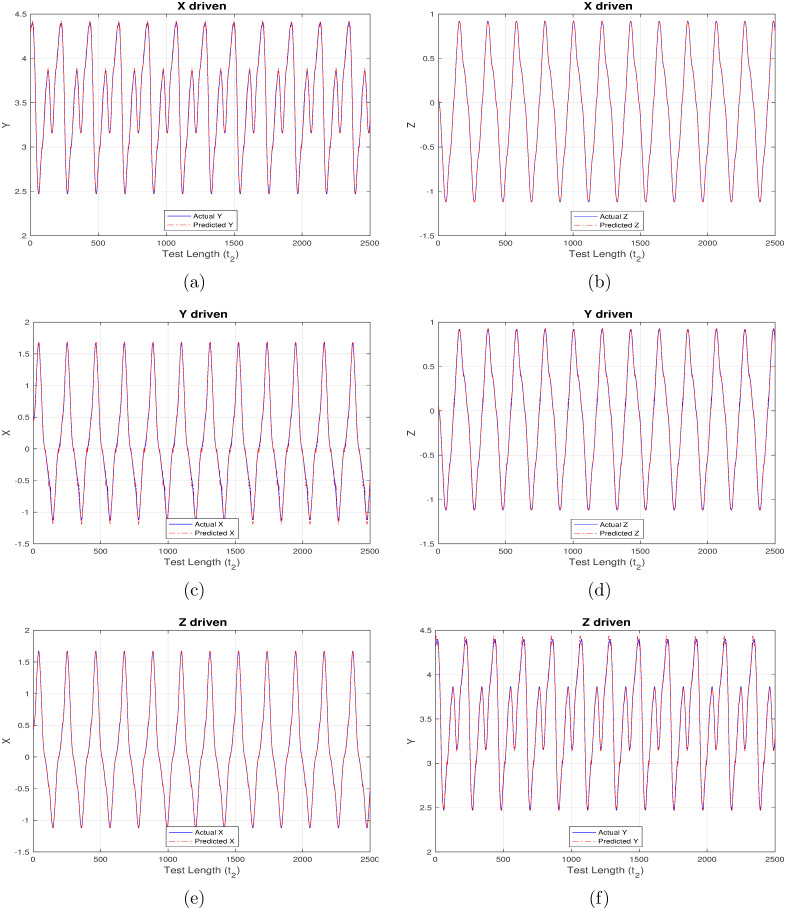
Controlled (time-delayed feedback) financial system component re-generation using “quadratic” activation. (a) Re-generating “investment demand” [Y] behavior with “interest rate” [X] as input (b) Re-generating “price index” [Z] behavior with “interest rate” [X] as input (c) Re-generating “interest rate”[X] behavior with “investment demand”[Y] as input (d) Re-generating “price index” [Z] behavior with “investment demand” [Y] as input (e) Re-generating “interest rate” [X] behavior with “price index” [Z] as input (f) Re-generating “investment demand” [Y] behavior with “price index” [Z] as input.

**Fig 11:**
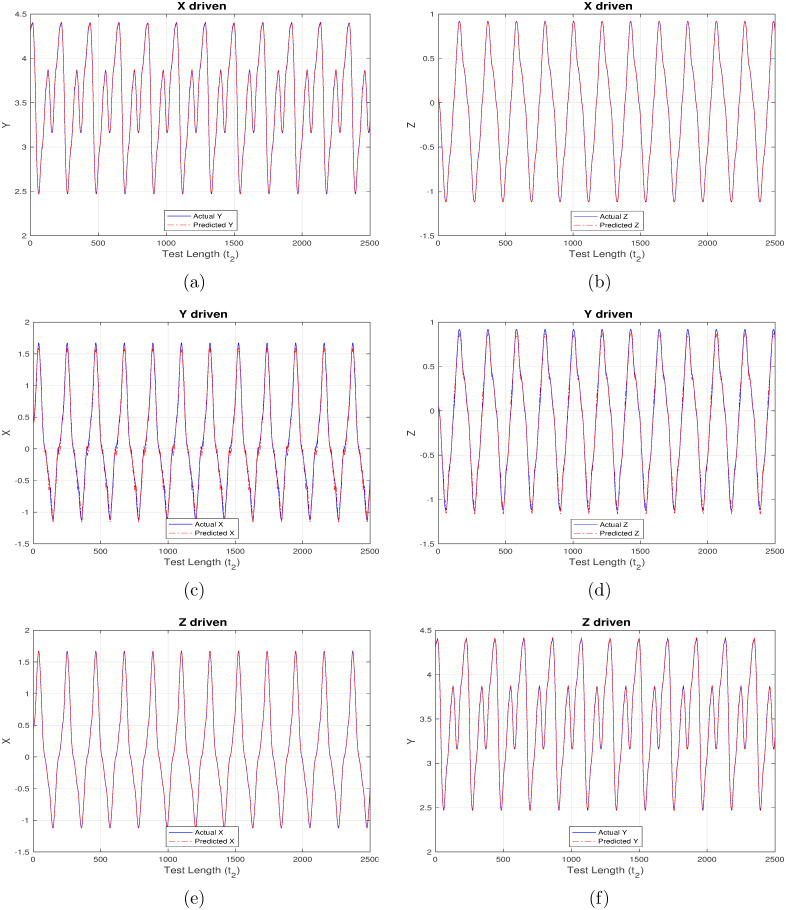
Controlled (time-delayed feedback) financial system component re-generation using “tanh” activation. (a) Re-generating “investment demand” [Y] behavior with “interest rate” [X] as input (b) Re-generating “price index” [Z] behavior with “interest rate” [X] as input (c) Re-generating “interest rate”[X] behavior with “investment demand”[Y] as input (d) Re-generating “price index” [Z] behavior with “investment demand” [Y] as input (e) Re-generating “interest rate” [X] behavior with “price index” [Z] as input (f) Re-generating “investment demand” [Y] behavior with “price index” [Z] as input.

Another set of inference results, depicted in Figs 8–11 are generated using the same control parameters defined in Tables 1–3 and “ControlledInput2” data which corresponds to the controlled, regularized financial system owing to the calculated induction of time-delayed feedback. Fig 8 maps to strictly linear activation, Fig 9 to strictly non-linear activation, Fig 10 to quadratic activation and Fig 11 to tanh activation functions. Each figure in turn comprises three set of plots, with each set corresponding to one component driving the reservoir and remaining two components being inferred from the model.

As opposed to the original chaotically behaved financial system, the strictly linear activation function is found to perform much better now, but still it is unable to match up with those having a non-linear component. As before, the linear activation function is found to behave exceedingly well while correlating X and Z components as shown in Fig 8b and 8e. Owing to the new regularized nature of the system, the introduction of Y component for driving the reservoir, or, during inference is also able to predict much better, thereby maintaining a high degree of congruency with the actual data. It is evident from Figs 9–11 that the X-driven, Y-driven or Z-driven reservoir systems could predict the remaining components with a high degree of accuracy. It is easily discernible that the overlap between actual and inferred components in far better in Figs 8–11, when compared to Figs 4–7. This confirms the theoretical expectation that prediction ratio must be far higher for systems displaying a regular and controlled behavior when compared to chaotic dynamical systems. This is further substantiated by the relatively low mean-square error (MSE) values mentioned in Tables 4 and 5.

**Table 5:** Mean square error (MSE) for chaotic system in Experiment set 2.

Activation Functions\Components	X-driven		Y-driven		Z-driven	
	Y	Z	X	Z	X	Y
**Linear**	0.41513	8.35E-03	0.66113	0.44551	0.033869	0.41797
**Strictly Non-linear**	0.073156	3.87E-03	0.65957	0.42509	0.014337	0.040206
**Quadratic**	0.037165	2.87E-03	0.67028	0.42386	0.010372	0.030827
**Tanh**	0.0078848	3.85E-04	0.64607	0.42246	0.0021148	0.016316

Comparing Tables 5 and 6, it is observed that the MSE values are relatively lower for a controlled system, implicitly referring to better approximation than a chaotic or dynamical system. Basis the above demonstrated results and resulting interpretations, it will be justified to mention reservoir computing as a highly versatile and low-cost scalable machine-learning technique which can be trained to infer the control states in systems requiring complex temporal processing capabilities. This should facilitate in helping it gain universal acceptance for efficiently reconstructing many rapidly evolving and complex system behaviors.

**Table 6:** Mean square error (MSE) for controlled system in Experiment set 2.

Activation Functions\Components	X-driven		Y-driven		Z-driven	
	Y	Z	X	Z	X	Y
**Linear**	0.15247	4.19E-03	0.33604	0.266	0.015512	0.18915
**Strictly Non-linear**	7.78E-04	1.70E-04	3.92E-03	1.17E-03	1.84E-04	1.50E-03
**Quadratic**	4.05E-04	9.72E-06	1.59E-03	3.38E-04	1.03E-04	6.94E-04
**Tanh**	4.54E-05	1.63E-06	3.22E-03	1.55E-3	9.93E-06	6.46E-05

## Conclusions

In the present work, we provide insights on the effectiveness of applying reservoir computing (RC) to complex and dynamical financial systems. Figs 2 and 3 generated using Experiment set 1 easily demonstrate the RC capability to forecast entire system behavior by executing in a feedback-only mode. The predicted values in Fig 2a–2d are well-aligned to the chaotic financial system behavioral patterns, but it becomes a near replica in Fig 3a–3d when the system is under the influence of a suitable time-delayed feedback. More importantly, the controlled system demonstrates a very high degree of memory retention by mirroring the target outputs for the entire test cycle having 2500 steps, beyond which deviation may be observed. Mean square error (MSE) values reported in Table 4 further conclude that a chaotic system under the influence of suitable time-delayed feedback is seen to predict much better and for a longer period when compared to same chaotic system devoid of time-delayed feedback.

Additionally, RC proves to be a robust and self-sufficient technique which can effectively re-generate parts of such non-linear systems when only limited future data is available for some system components. This is evident from Figs 4–11 generated as part of Experiment set 2 where in the economic indices are inferred from a limited set of continuously available system information. The efficiency of this approach is further corroborated by the presence of low MSE values as depicted in Tables 5 and 6. Moreover, the results conclude that activation functions having a non-linear component have a better inference capability compared to strictly non-linear functions for such dynamical systems. In our case, tanh is seen to outperform purely non-linear, quadratic and purely linear activation functions. Attaining such a controlled accuracy enables the government and other policy making bodies to adopt measures even when the scope of interference is limited, in addition to providing the capability of taking timely, well-informed and globally beneficial decisions.

As a next step, it will be beneficial to observe the same financial system behavior by tuning all the feedback strength coefficients (*f*_1_, *f*_2_, *f*_3_), along with the application of multi-layered, or, deep reservoir networks. This should help envisage the complexity and computation trade-off existing between single and multi-layered reservoirs for such non-linear and complex dynamical systems.
